# Potential Survival Benefit of Adjuvant Chemotherapy in Stage IV Intrahepatic Cholangiocarcinoma: A Multicenter, Stage‐Stratified Analysis

**DOI:** 10.1002/ags3.70087

**Published:** 2025-08-31

**Authors:** Hisashi Kosaka, Masaki Ueno, Hiroji Shinkawa, Yusuke Yamamoto, Masahiko Kinoshita, Koji Komeda, Tsukasa Aihara, Satoshi Yasuda, Haruki Mori, Masaki Kaibori

**Affiliations:** ^1^ Department of Hepatobiliary Surgery Kansai Medical University Hirakata Japan; ^2^ Department of Surgery Osaka Minami Medical Center Osaka Japan; ^3^ Department of Hepato‐Biliary‐Pancreatic Surgery Osaka Metropolitan University Osaka Japan; ^4^ Department of Digestive Surgery Kyoto Prefectural University of Medicine Kyoto Japan; ^5^ Department of Surgery Osaka Medical and Pharmaceutical University Osaka Japan; ^6^ Department of Surgery Meiwa Hospital Nishinomiya Hyogo Japan; ^7^ Department of Surgery Nara Medical University Nara Japan; ^8^ Department of Surgery Shiga University of Medical Science Otsu Shiga Japan

**Keywords:** adjuvant, chemotherapy, fluoropyrimidines, intrahepatic cholangiocarcinoma

## Abstract

**Background:**

The survival benefit of adjuvant chemotherapy (AC) in intrahepatic cholangiocarcinoma (ICC) remains uncertain, particularly in advanced‐stage disease.

**Methods:**

We retrospectively analyzed 480 patients who underwent curative‐intent hepatic resection for ICC at eight institutions between 2006 and 2023. Patients were stratified by receipt of AC, and survival outcomes were compared across LCSGJ stages. Multivariable Cox regression was used to identify prognostic factors.

**Results:**

Among 480 patients, 206 received AC. While AC did not significantly improve survival in stage I–III disease, it was associated with significantly longer overall survival (median 25.5 vs. 17.1 months, *p* = 0.008) and recurrence‐free survival (median 10.3 vs. 6.0 months, *p* = 0.010) in stage IV patients. Multivariable analysis in stage IV revealed that AC independently reduced the risk of death (HR 0.540, *p* = 0.020), while poor liver function, severe postoperative complications, tumor size, and lymph node metastasis were adverse prognostic factors. Among AC regimens, S‐1 demonstrated significantly longer OS (69.3 vs. 17.1 months, *p* = 0.001) and RFS (9.6 vs. 6.0 months, *p* = 0.015) compared with no AC, whereas other regimens did not show statistically significant benefits.

**Conclusions:**

Adjuvant chemotherapy was associated with improved survival in patients with resected stage IV ICC. Among available regimens, S‐1 appeared to contribute to this benefit. These findings support the use of AC in advanced ICC and suggest that S‐1 may play a potential role, warranting further prospective validation. Stage‐specific treatment planning may be essential to optimize outcomes.

## Graphical abstract

This multicenter study suggests that adjuvant chemotherapy may improve survival in stage IV intrahepatic cholangiocarcinoma after resection, with S‐1 showing a potential survival advantage.

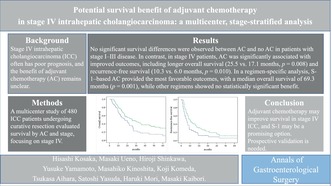

## 1 Introduction

Intrahepatic cholangiocarcinoma (ICC) is an aggressive malignancy originating from the intrahepatic bile ducts and accounts for approximately 10%–15% of all primary liver cancers [1]. Its global incidence has been steadily increasing, and the long‐term prognosis remains poor [2]. Despite advances in surgical techniques and perioperative care, the 5‐year overall survival (OS) following curative‐intent liver resection remains low, ranging from 20% to 40%, primarily due to high recurrence rates. These limitations underscore the urgent need for effective adjuvant treatment strategies.

Although adjuvant chemotherapy (AC) is commonly used in gastrointestinal malignancies, its survival benefit in ICC remains controversial. Most published studies have evaluated AC in biliary tract cancer (BTC) as a whole, grouping ICC with extrahepatic cholangiocarcinoma and gallbladder cancer [3, 4]. Consequently, the efficacy of AC specifically for ICC has not been clearly established. A recent meta‐analysis suggested a potential survival benefit of adjuvant chemotherapy in resectable intrahepatic cholangiocarcinoma; however, definitive conclusions remain limited due to the heterogeneity of study populations and the small size of ICC‐specific cohorts [5]. Among prospective trials, the BILCAP study demonstrated a survival benefit of adjuvant capecitabine over observation in patients with biliary tract cancer (BTC) in the per‐protocol analysis, although the primary intention‐to‐treat analysis did not reach statistical significance [6]. Similarly, the ASCOT trial showed a significant improvement in overall survival with S‐1 in resected BTC, although its effect on relapse‐free survival was not statistically significant [7]. Notably, both studies included mixed BTC populations, and ICC‐specific subgroup analyses lacked sufficient statistical power to draw firm conclusions.

To address this evidence gap, we conducted a multicenter retrospective study focusing exclusively on patients with resected ICC. Our objective was to evaluate the potential survival benefit of adjuvant chemotherapy by comparing overall survival between patients who received AC and those who did not. In addition, we performed a stage‐specific analysis to determine whether the impact of AC varied according to pathological stage.

## 2 Materials and Methods

### 2.1 Patients

**FIGURE 1:**
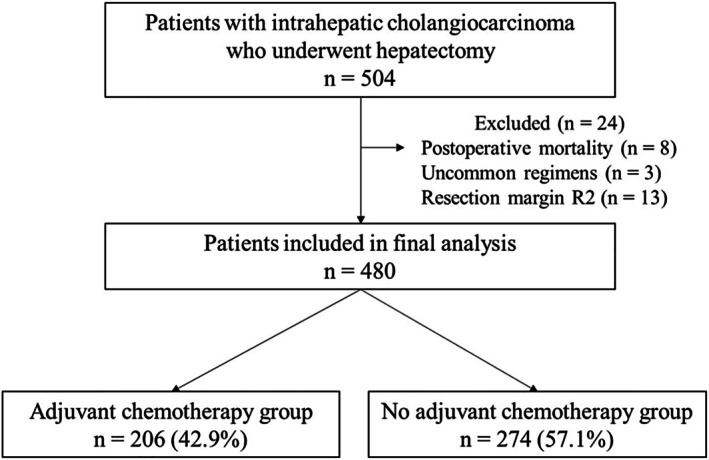
Study design and patient grouping based on adjuvant chemotherapy status. This flowchart outlines the study design and patient classification. A total of 504 patients with intrahepatic cholangiocarcinoma who underwent curative‐intent hepatectomy across seven institutions were retrospectively reviewed. After excluding ineligible cases, patients were divided into two groups according to postoperative adjuvant chemotherapy status: Those who received adjuvant chemotherapy and those who did not.

This multicenter retrospective study included patients who underwent curative‐intent hepatic resection for ICC between 2006 and 2023 at eight institutions in Japan. The diagnosis of ICC was histologically confirmed in all cases. Among 504 initially identified patients, 8 were excluded due to postoperative mortality and 3 due to receipt of uncommon adjuvant chemotherapy regimens, and 13 due to R2 resection margins, leaving a final cohort of 480 patients for analysis (Figure 1). Of these, 206 patients received AC, while the remaining 274 patients did not. Postoperative surveillance was performed through outpatient visits, with follow‐up evaluations including imaging studies and blood tests such as tumor marker assessments. Follow‐up intervals were generally every 3 to 6 months during the first 2 years and every 6 to 12 months thereafter, in accordance with institutional practice. Clinical and pathological data were retrospectively collected from medical records at each participating institution. This study was approved by the institutional review board of Kansai Medical University (Approval number: 2023320) and conducted in accordance with the principles of the Declaration of Helsinki. Informed consent was obtained using an opt‐out approach approved by each institution.

### 2.2 Adjuvant Chemotherapy

AC regimens were administered according to institutional protocols and were typically continued for 6 months. The median interval from surgery to the initiation of chemotherapy was 1.6 months (IQR 1.10–2.30). Gemcitabine monotherapy was given intravenously at 1000 mg/m^2^ on Days 1, 8, and 15 of a 28‐day cycle. The gemcitabine plus cisplatin (GC) regimen consisted of gemcitabine (1000 mg/m^2^) and cisplatin (25 mg/m^2^) administered intravenously on Days 1 and 8 of a 21‐day cycle. For gemcitabine plus S‐1 (GS), gemcitabine was administered intravenously on Days 1 and 8, and S‐1 was taken orally twice daily on Days 1–14 of a 21‐day cycle. The gemcitabine, cisplatin, and S‐1 (GCS) regimen involved intravenous administration of gemcitabine (1000 mg/m^2^) and cisplatin (25 mg/m^2^) on Day 1 of a 14‐day cycle, with oral S‐1 given twice daily on Days 1–7. In the S‐1 monotherapy regimen, S‐1 was administered orally twice daily for 28 days followed by a 14‐day rest, constituting a 6‐week cycle. For all S‐1–containing regimens, the daily dose of S‐1 was determined based on body surface area (BSA): 80 mg/day for BSA < 1.25 m^2^, 100 mg/day for BSA 1.25–1.5 m^2^, and 120 mg/day for BSA ≥ 1.5 m^2^. Relative dose intensity (RDI) was calculated as the ratio of the actual delivered dose intensity (mg/m^2^ per week) to the planned dose intensity, expressed as a percentage. RDI was assessed over the first 6 months from the initiation of adjuvant chemotherapy.

### 2.3 Statistical Analysis

Data are presented as numbers with percentages for categorical variables and as medians with interquartile ranges (IQRs) for continuous variables. The Mann–Whitney *U* test was used for continuous variables. The Fisher's exact test was used for categorical variables. OS and recurrence‐free survival (RFS) were estimated using the Kaplan–Meier method and compared using the log‐rank test. OS was defined as the time from the date of surgery to the date of death or last confirmed survival, and RFS as the time from the date of surgery to the date of recurrence or last follow‐up without recurrence. Patients without an event (recurrence or death) were censored at the date of last follow‐up or last confirmed survival. Time to adjuvant chemotherapy was calculated from the date of surgery to the date of chemotherapy initiation. To evaluate prognostic factors for overall survival, a multivariable analysis was performed using the Cox proportional hazards model with the enter method. All clinically relevant variables were simultaneously included in the model. Hazard ratios (HRs) with corresponding 95% confidence intervals (CIs) and *p* values were calculated. A *p*‐value of < 0.05 was considered statistically significant. All statistical analyses were performed using IBM SPSS Statistics for Windows, version 22.0 (IBM Japan Ltd., Tokyo, Japan).

## 3 Results

### 3.1 Background Characteristics

Preoperative and intraoperative characteristics stratified by adjuvant chemotherapy status are summarized in Table 1. Patients who received adjuvant chemotherapy tended to have better preserved liver function, as reflected by a lower prevalence of hepatitis C virus infection (7.3% vs. 19.0%, *p* < 0.001), higher platelet counts (20.9 × 10^4^/μL vs. 19.1 × 10^4^/μL, *p* = 0.029), lower FIB‐4 index (1.95 vs. 2.23, *p* < 0.001), and lower indocyanine green retention rates (9.5% vs. 10.6%, *p* = 0.003), compared to patients who did not receive adjuvant therapy. The extent of hepatectomy was also greater in the adjuvant chemotherapy group, which was associated with longer operative time (376.5 vs. 332.0 min, *p* < 0.001) and increased intraoperative blood loss (607.5 vs. 413.0 mL, *p* = 0.002). Lymph node metastasis was more frequent in the adjuvant chemotherapy group than in the no adjuvant group (25.2% vs. 13.5%, *p* = 0.001). There were no significant differences between the two groups in other perioperative parameters, including R0 resection rate (87.9% vs. 85.0%, *p* = 0.373) and incidence of severe postoperative complications (Clavien–Dindo ≥ IIIa: 24.3% vs. 25.2%, *p* = 0.819).

**TABLE 1:** Clinicopathological characteristics of patients with intrahepatic cholangiocarcinoma undergoing hepatectomy, stratified by adjuvant chemotherapy status.

Parameters	*n* (%) or median (IQR)		*p*
	Adjuvant chemotherapy, *n* = 206	No adjuvant chemotherapy, *n* = 274	
Age	71.0 (63.0–75.0)	72.0 (66.0–77.0)	0.005
Gender, male	131 (63.6)	192 (70.1)	0.134
Body mass index	23.2 (21.1–25.3)	23.0 (20.0–25.1)	0.157
Hepatitis B virus infection	26 (12.6)	38 (13.9)	0.691
Hepatitis C virus infection	15 (7.3)	52 (19.0)	< 0.001
Diabetes mellitus	51 (24.8)	69 (25.2)	0.915
Total bilirubin, mg/dL	0.7 (0.5–0.9)	0.7 (0.5–0.9)	0.847
Albumin, g/dL	4.2 (3.8–4.5)	4.1 (3.8–4.3)	0.069
AST, U/L	27.0 (22.0–32.3)	27.0 (22.0–37.0)	0.173
ALT, U/L	23.5 (17.0–32.3)	22.0 (15.8–33.3)	0.280
Platelet count, ×10^4^μL	20.9 (16.7–25.8)	19.1 (14.3–24.2)	0.029
CRP, mg/dL	0.17 (0.08–0.39)	0.14 (0.05–0.38)	0.120
ICG, %	9.5 (6.3–13.0)	10.6 (8.0–14.8)	0.003
ALBI score	−2.85 (−3.08 – −2.53)	−2.81 (−3.02 – −2.53)	0.079
FIB‐4 index	1.95 (1.34–2.51)	2.23 (1.67–3.13)	< 0.001
CEA, ng/mL	3.0 (1.7–5.6)	2.8 (1.9–4.4)	0.302
CA19‐9, U/mL	54.2 (14.8–337.4)	31.8 (9.9–204.5)	0.010
Laparoscopic approach	45 (21.8)	73 (26.6)	0.227
Range of hepatectomy			
Segmentectomy or less	35 (17.0)	88 (32.1)	< 0.001
Sectionectomy	23 (11.2)	43 (15.7)	
Bi‐sectionectomy	137 (66.5)	137 (50.0)	
Tri‐sectionectomy	11 (5.3)	6 (2.2)	
Operative time, min	376.5 (293.8–503.3)	332.0 (252.0–438.8)	< 0.001
Blood loss, mL	607.5 (300.0–1138.3)	413.0 (140.0–931.3)	0.002
Blood transfusion	53 (25.9)	61 (22.3)	0.361
Clavien–Dindo ≥ IIIa	50 (24.3)	69 (25.2)	0.819
Resection margin			
R0	181 (87.9)	233 (85.0)	0.373
R1	25 (12.1)	41 (15.0)	
Lymph node metastasis	52 (25.2)	37 (13.5)	0.001
LCSGJ stage			
I	10 (4.9)	23 (8.4)	< 0.001
II	45 (21.8)	96 (35.0)	
III	80 (38.8)	102 (37.2)	
IVA	54 (26.2)	45 (16.4)	
IVB	17 (8.3)	8 (2.9)	

Abbreviations: ALBI, albumin–bilirubin; ALT, alanine aminotransferase; AST, aspartate aminotransferase; CA19‐9, carbohydrate antigen 19–9; CEA, carcinoembryonic antigen; CRP, C‐reactive protein; FIB‐4, fibrosis‐4 index; ICG, indocyanine green; IQR, interquartile range; LCSGJ, Liver Cancer Study Group of Japan.

### 3.2 Distribution of Adjuvant Chemotherapy by Stage

The distribution of adjuvant chemotherapy regimens varied significantly according to pathological stage (*p* < 0.001, Table 2). Among patients with stage I and II disease, 69.7% and 68.1%, respectively, received no adjuvant chemotherapy. In contrast, the proportion of patients without adjuvant therapy declined to 56.0% in stage III and 42.7% in stage IV. The use of multi‐agent regimens increased with advancing stage: GC was administered in 17.0% of stage III and 12.9% of stage IV patients; GS was used in 9.7% of stage IV patients, but in ≤ 2.8% of earlier‐stage cases; and GCS was exclusively employed in stage IV disease (4.0%). In contrast, S‐1 monotherapy was more evenly distributed across all stages, ranging from 17.0% to 21.2%. The median RDI varied by regimen, with S‐1 and GC showing moderate intensities (83.3% and 80.0%, respectively), whereas gemcitabine monotherapy and GS achieved 100% median RDI. GCS also maintained a relatively high RDI (99.2%). There was a statistically significant difference in RDI among regimens (*p* = 0.020). Similarly, the median time from surgery to AC initiation was 1.6 months (IQR 1.1–2.3) with no significant difference among regimens (*p* = 0.408). These data indicate that most patients were able to maintain planned dosing and initiate AC within a comparable timeframe, suggesting generally good treatment adherence.

**TABLE 2:** Distribution of adjuvant chemotherapy regimens by stage defined by the Liver Cancer Study Group of Japan and corresponding overall relative dose intensity.

	*n* (%)				*p*	Median (IQR)
	Stage I	Stage II	Stage III	Stage IV		RDI
No AC	23 (69.7)	96 (68.1)	102 (56.0)	53 (42.7)	< 0.001	—
S‐1	7 (21.2)	24 (17.0)	34 (18.7)	26 (21.0)		83.3 (66.4–100.0)
GC	3 (9.1)	10 (7.1)	31 (17.0)	16 (12.9)		80.0 (66.4–90.0)
GEM	0 (0.0)	7 (5.0)	10 (5.5)	12 (9.7)		100.0 (80.0–100.0)
GS	0 (0.0)	4 (2.8)	4 (2.2)	12 (9.7)		100.0 (55.0–100.0)
GCS	0 (0.0)	0 (0.0)	1 (0.5)	5 (4.0)		99.2 (66.7–NE)

*Note:* Values are presented as number of patients (percentage within each stage). RDI represents the overall average for each regimen.

Abbreviations: GC, gemcitabine plus cisplatin; GCS, gemcitabine plus cisplatin plus S‐1; GEM, gemcitabine monotherapy; GS, gemcitabine plus S‐1; IQR, interquartile range; NE, not estimable; No AC, no adjuvant chemotherapy; RDI, relative dose intensity; S‐1, oral fluoropyrimidine.

### 3.3 Patient Survival Stratified by Adjuvant Chemotherapy Status and LCSGJ Stage

**FIGURE 2:**
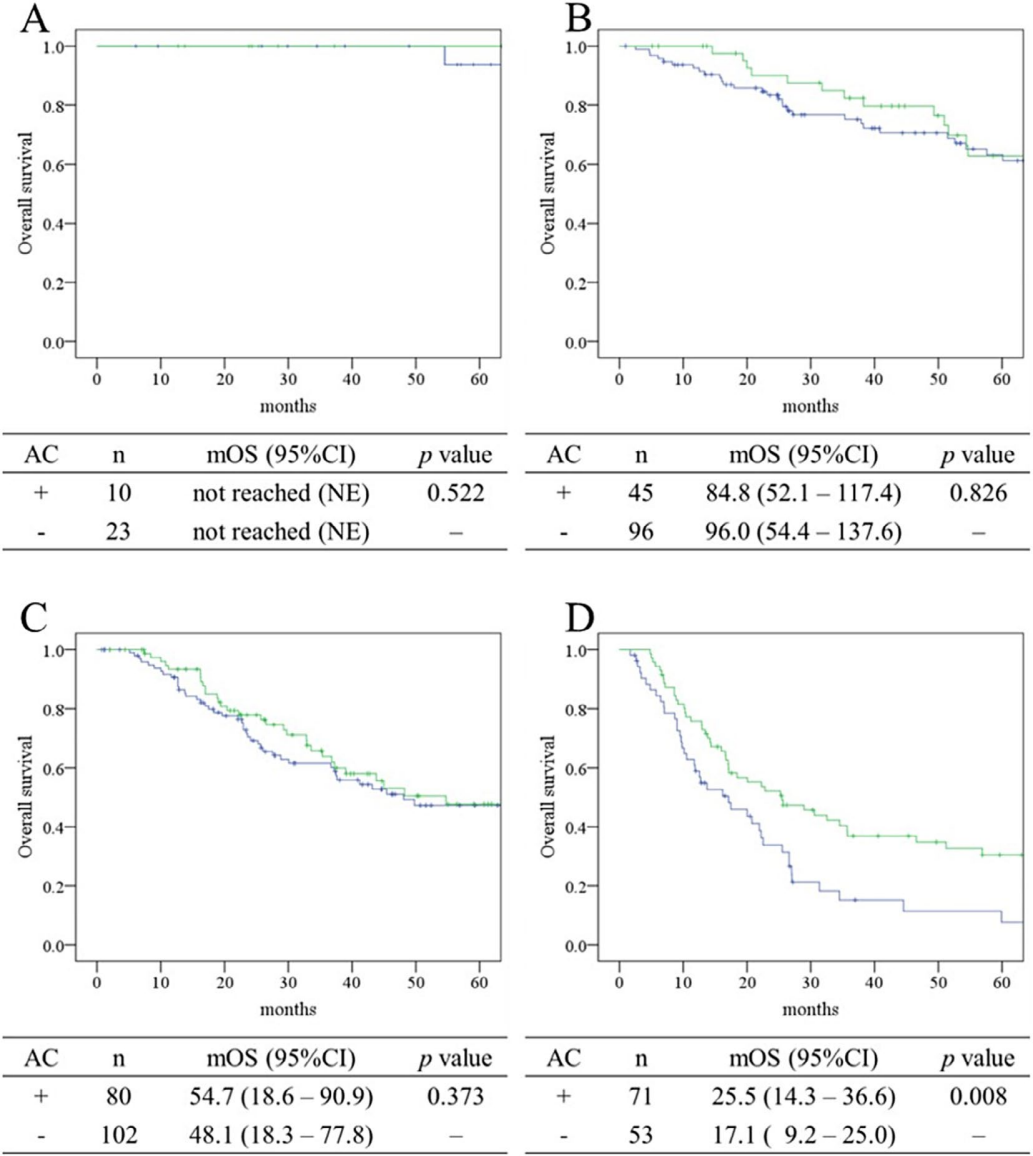
Comparison of overall survival by adjuvant chemotherapy status and stage defined by the Liver Cancer Study Group of Japan. Overall survival is presented using Kaplan–Meier curves for each stage defined by the Liver Cancer Study Group of Japan (A: Stage I, B: Stage II, C: Stage III, D: Stage IVA and IVB). The green line represents patients who received adjuvant chemotherapy, and the blue line represents those who did not. Median overall survival is shown in months. AC, adjuvant chemotherapy; CI, confidence interval; mOS, median overall survival.

OS curves stratified by adjuvant chemotherapy status (AC vs. no AC) according to LCSGJ stage are shown in Figure 2. In stages I–III (panels A–C), overall survival did not differ significantly between patients who received adjuvant chemotherapy and those who did not. In contrast, patients with stage IVA/IVB disease (panel D) who received AC demonstrated markedly improved outcomes, with a median OS of 25.5 months compared to 17.1 months in the no AC group and 5‐year OS rates of 30.5% vs. 7.6%, respectively (*p* = 0.008).

**FIGURE 3:**
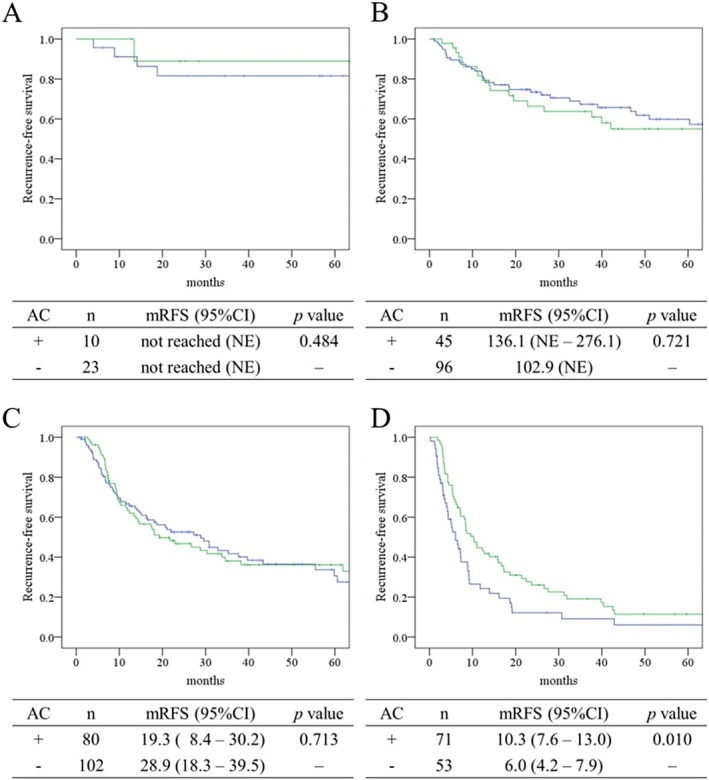
Comparison of recurrence‐free survival by adjuvant chemotherapy status and stage defined by the Liver Cancer Study Group of Japan. Recurrence‐free survival is presented using Kaplan–Meier curves for each stage defined by the Liver Cancer Study Group of Japan (A: Stage I, B: Stage II, C: Stage III, D: Stage IVA and IVB). The green line represents patients who received adjuvant chemotherapy, and the blue line represents those who did not. Median recurrence‐free survival is shown in months. AC, adjuvant chemotherapy; CI, confidence interval; mRFS, median recurrence‐free survival.

RFS outcomes, illustrated in Figure 3, showed a comparable trend. In stages I–III (panels A–C), no statistically significant differences in recurrence‐free survival were observed between the groups. However, in patients with stage IVA/IVB (panel D), adjuvant chemotherapy was associated with prolonged RFS, with a median duration of 10.3 months in the AC group and 6.0 months in the no AC group. The corresponding 5‐year RFS rates were 11.5% and 6.1%, respectively (*p* = 0.010).

### 3.4 Multivariable Analysis for Overall Survival in Stage IV Patients

Prognostic factors for overall survival in stage IV ICC were identified by multivariable Cox regression analysis (Table 3). Several variables were found to be significantly associated with prognosis. Poor liver function, as assessed by higher ALBI scores, was independently associated with worse overall survival (HR 1.960, 95% CI 1.057–3.634, *p* = 0.033). Severe postoperative complications (Clavien–Dindo grade ≥ IIIa) were also an adverse prognostic factor (HR 1.761, 95% CI 1.070–2.897, *p* = 0.026). Lymph node metastasis showed a trend toward worse survival but did not reach statistical significance (HR 1.704, 95% CI 0.976–2.975, *p* = 0.061). The use of adjuvant chemotherapy was significantly associated with improved survival in this advanced‐stage subgroup (HR 0.540, 95% CI 0.322–0.907, *p* = 0.020). Other variables such as tumor diameter, CA19‐9 level, extent of hepatectomy, and resection margin status did not show a significant association with overall survival.

**TABLE 3:** Multivariable Cox regression analysis for overall survival in patients with stage IV intrahepatic cholangiocarcinoma.

Variable	B	SE	Wald	*p*	HR	95% CI
Age	−0.005	0.014	0.119	0.730	0.995	0.968–1.023
Hepatitis C virus infection	−0.163	0.406	0.160	0.689	0.850	0.384–1.884
ALBI score	0.673	0.315	4.559	0.033	1.960	1.057–3.634
FIB‐4 index	−0.050	0.064	0.602	0.438	0.951	0.839–1.079
CA19‐9	0.053	0.090	0.340	0.560	1.054	0.883–1.258
Extent of hepatectomy	−0.040	0.179	0.049	0.825	0.961	0.677–1.365
Clavien–Dindo ≥ IIIa	0.566	0.254	4.960	0.026	1.761	1.070–2.897
Resection margin status	0.183	0.302	0.369	0.544	1.201	0.665–2.170
Tumor diameter	0.006	0.004	2.346	0.126	1.006	0.998–1.014
Lymph node metastasis	0.533	0.284	3.511	0.061	1.704	0.976–2.975
Adjuvant chemotherapy	−0.615	0.264	5.436	0.020	0.540	0.322–0.907

*Note:* Multivariable analysis in the entire cohort is shown in Table S1.

Abbreviations: ALBI, albumin–bilirubin score; B, regression coefficient; CA19‐9, carbohydrate antigen 19‐9; CI, confidence interval; FIB‐4, fibrosis‐4; HR, hazard ratio; SE, standard error; Wald, Wald chi‐squared statistic.

To complement this analysis, we also performed a multivariable Cox regression for the entire cohort (*n* = 480). In this analysis, ALBI score, CA19‐9 level, extent of hepatectomy, postoperative complications, tumor size, and lymph node metastasis were significantly associated with overall survival. Although adjuvant chemotherapy showed a trend toward improved survival, the difference did not reach statistical significance (*p* = 0.092). The results are summarized in Table S1.

### 3.5 Subgroup Analysis of Stage IV Disease by Lymph Node Status and IVA/IVB Subclassification

Clinicopathological features of IVA and IVB, as well as AC/no AC–stratified details for these stages, are summarized in Tables S2 and S3. These tables show marked differences in nodal involvement and margin status between IVA and IVB, while baseline characteristics were generally comparable between AC and no AC groups. Given this heterogeneity, subgroup analyses were performed according to lymph node status and IVA/IVB subclassification. In the N0 subgroup, the median OS with AC was 56.9 months, but this difference did not reach statistical significance (*p* = 0.070). In the N1 subgroup, AC was associated with significantly longer median OS compared with no AC (22.8 vs. 13.7 months, *p* = 0.029) (Figure S1). For IVA/IVB subclassification, both stage IVA and stage IVB disease showed a trend toward longer survival with AC compared to no AC; however, neither difference reached statistical significance (stage IVA: median OS 32.5 vs. 17.5 months, *p* = 0.070; stage IVB: 11.2 vs. 9.0 months, *p* = 0.288) (Figure S2, Table S2).

### 3.6 Comparison of Survival by Adjuvant Chemotherapy Regimen in Stage IV Patients

**FIGURE 4:**
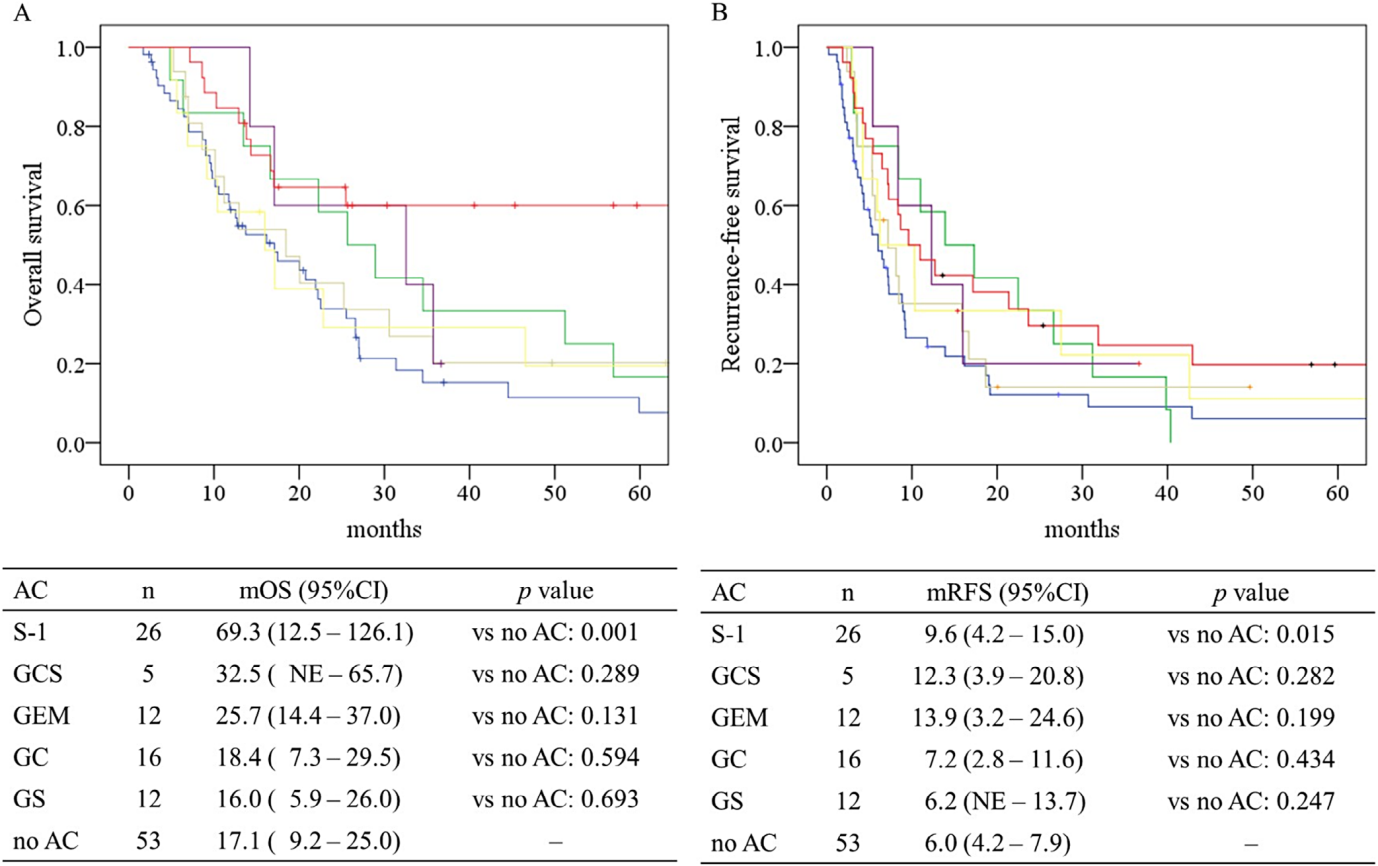
Overall and recurrence‐free survival by adjuvant chemotherapy regimen in stage IV intrahepatic cholangiocarcinoma. Kaplan–Meier curves are shown for patients with stage IV intrahepatic cholangiocarcinoma stratified by adjuvant chemotherapy regimen. Panel A shows overall survival, and Panel B shows recurrence‐free survival. The curves are color‐coded by regimen as follows: Patients who did not receive adjuvant chemotherapy are shown in blue, those treated with gemcitabine monotherapy (GEM) in green, gemcitabine plus cisplatin (GC) in beige, gemcitabine plus S‐1 (GS) in yellow, gemcitabine plus cisplatin plus S‐1 (GCS) in purple, and S‐1 monotherapy (S) in red. Median overall survival and median recurrence‐free survival are shown in months. AC, adjuvant chemotherapy; AC, adjuvant chemotherapy; CI, confidence interval; GC, gemcitabine plus cisplatin; GCS, gemcitabine plus cisplatin plus S‐1; GEM, gemcitabine monotherapy; GS, gemcitabine plus S‐1; mOS, median overall survival; mRFS, median recurrence‐free survival; NE, not estimable; S‐1, oral fluoropyrimidine.

Figure 4 presents Kaplan–Meier survival curves for overall survival (OS; panel A) and recurrence‐free survival (RFS; panel B) stratified by adjuvant chemotherapy regimen in patients with stage IV ICC. In terms of OS, patients who received S‐1–based adjuvant chemotherapy showed significantly improved survival compared with those who did not receive adjuvant chemotherapy (median OS: 69.3 months, 95% CI 12.5–126.1, *p* = 0.001). The GCS (median OS: 32.5 months, 95% CI not estimable—65.7, *p* = 0.289) and GEM monotherapy groups (25.7 months, 95% CI 14.4–37.0, *p* = 0.131) also showed longer median survival than the no adjuvant chemotherapy group, but these differences were not statistically significant. A similar pattern was observed for RFS. The S‐1 group had a significantly longer median RFS compared to no adjuvant chemotherapy (9.6 months, 95% CI 4.2–15.0, *p* = 0.015), whereas the GCS (12.3 months, 95% CI 3.9–20.8, *p* = 0.282) and GEM (13.9 months, 95% CI 3.2–24.6, *p* = 0.199) groups had longer median RFS than no adjuvant chemotherapy but without statistical significance.

## 4 Discussion

This multicenter retrospective study focused on the role of AC in patients with ICC, with particular attention to stage‐specific outcomes. While no clear survival benefit of AC was observed in patients with stage I–III disease, a significant improvement was evident in patients with stage IV disease, suggesting a possible stage‐dependent effect of AC, as it significantly improved both RFS (median 10.3 vs. 6.0 months, *p* = 0.010) and OS (median 25.5 vs. 17.5 months, *p* = 0.013) in this subgroup, likely through suppression of postoperative recurrence. This finding underscores the importance of tailoring postoperative treatment strategies based on disease stage and provides supportive evidence for the use of AC in advanced ICC, consistent with findings from BILCAP and ASCOT [6, 7]. In contrast, among patients with stage I–III disease, AC did not confer a significant survival benefit, suggesting that its routine use may be unnecessary in lower‐risk cases. These results highlight the need for careful risk stratification and individualized treatment planning. Compared with previous reports that combined multiple BTC subtypes, this study's focus on ICC alone—and further stratification by pathological stage—provides a more granular assessment of adjuvant treatment efficacy. The observed benefit in stage IV ICC may contribute to a deeper understanding of the role of AC in advanced disease.

Among the regimens analyzed, S‐1 monotherapy demonstrated a significant improvement in both OS (median 69.3 vs. 17.5 months, *p* = 0.002) and RFS (median 9.6 vs. 6.0 months, *p* = 0.015) compared to no adjuvant chemotherapy. This benefit was particularly evident in patients with stage IV ICC, highlighting the potential role of S‐1 in advanced cases. These findings are in agreement with previous studies, including the ASCOT trial, reporting the efficacy of S‐1 in resected biliary tract cancer [7, 8]. S‐1 was developed as an oral fluoropyrimidine that combines tegafur, a prodrug of 5‐FU, with two modulators: gimeracil, which inhibits 5‐FU degradation, and oteracil, which reduces gastrointestinal toxicity [9]. This design enhances antitumor activity while maintaining tolerability, supporting its clinical utility in postoperative settings. Consistent with the BILCAP trial, which did not demonstrate a significant survival benefit of GEM monotherapy, our study also failed to show a statistically significant advantage for GEM in stage IV ICC [10]. Although S‐1 appeared to outperform combination regimens such as GCS, GS, or GC in the present analysis, this finding should be interpreted with caution. The number of patients receiving each combination regimen—particularly GCS—was relatively small, and the median follow‐up period was limited, both of which may have reduced the power to detect true differences. In fact, GCS showed a trend toward improved survival in Figure 4, suggesting that with a larger sample size and longer observation, the outcomes might differ. Furthermore, the distribution of second‐line treatment modalities, including local therapy (surgery, radiofrequency ablation, or transarterial chemoembolization), chemotherapy, or best supportive care, did not differ significantly among AC regimens (*p* = 0.511). However, gemcitabine monotherapy and GS were predominantly administered in earlier periods, whereas S‐1 was more commonly used in recent years. Therefore, the improved OS observed in the S‐1 group may partly reflect the availability of more effective second‐line options, such as triplet chemotherapy regimens, in the modern treatment era. This temporal trend could also explain the discrepancy observed between OS and RFS: while RFS is less likely to be influenced by subsequent treatments, OS may have been prolonged in the S‐1 group due to more effective post‐recurrence therapies available in recent years. Taken together, this temporal effect, combined with the limited sample size and follow‐up period, may have influenced the observed survival differences and should be considered when interpreting the present findings. Thus, caution is warranted when attributing the OS advantage solely to the AC regimen.

Multivariable analysis identified adjuvant chemotherapy as an independent prognostic factor for overall survival in patients with stage IV ICC, significantly reducing the risk of death by approximately 39% (HR 0.612, 95% CI 0.381–0.984, *p* = 0.043), supporting its survival benefit in advanced disease. In contrast, poor liver function (ALBI score), severe postoperative complications (Clavien–Dindo grade ≥ IIIa), larger tumor size, and lymph node metastasis were also independently associated with worse prognosis. Among these, a higher ALBI score was significantly linked to increased mortality risk (HR 1.935, 95% CI 1.100–3.404, *p* = 0.022), consistent with previous studies demonstrating the prognostic value of ALBI in ICC and other hepatobiliary malignancies [11, 12]. Furthermore, severe postoperative complications independently predicted poor survival (HR 1.863, 95% CI 1.163–2.984, *p* = 0.010). Previous studies have also demonstrated that postoperative complications negatively impact long‐term survival following hepatic resection for ICC and other liver malignancies [13, 14, 15]. Delays in the initiation of adjuvant chemotherapy (AC) have been associated with worse outcomes; in colorectal cancer, previous studies have shown that starting AC within 8 weeks postoperatively is associated with improved survival [16]. In our study, the median time to AC initiation was significantly longer in patients with severe postoperative complications compared to those without (9.7 vs. 5.9 weeks, *p* < 0.001). These findings suggest that delays in AC initiation due to perioperative morbidity may have adversely affected prognosis, underscoring the importance of careful perioperative management and timely postoperative treatment planning.

## 5 Limitations

Several limitations should be considered. First, the retrospective design of this multicenter study may have introduced selection bias and unmeasured confounding, despite statistical adjustment using multivariable analysis. Second, AC regimens were not standardized across institutions, with variability in drug selection, dose intensity, duration, and timing of initiation, which may have affected outcomes. Third, although the analysis focused on stage IV disease, the number of patients in each substaged group (IVA and IVB) was limited, and these were analyzed together, potentially obscuring subgroup‐specific effects. Similarly, subgroup analyses stratified by lymph node status or IVA/IVB subclassification were underpowered due to the small sample size, and no statistically robust conclusions could be drawn; these findings should therefore be interpreted with caution. Fourth, differences in follow‐up protocols and perioperative management across institutions may have introduced heterogeneity. Fifth, data on treatment adherence, adverse events, and completion rates were incomplete, limiting the ability to evaluate regimen‐specific tolerability and feasibility. However, treatment adherence and completion were indirectly assessed using relative dose intensity (RDI), which was consistently high (≥ 80%) across regimens, suggesting generally good feasibility.

## 6 Conclusions

This multicenter study suggests that AC may improve survival in patients with stage IV ICC following curative‐intent resection. Our findings support the use of AC in advanced ICC and indicate that S‐1 could be a beneficial option.

## Author Contributions

**Hisashi Kosaka:** conceptualization, methodology, software, data curation, supervision, formal analysis, validation, investigation, funding acquisition, visualization, project administration, resources, writing – original draft, writing – review and editing. **Masaki Ueno:** data curation. **Hiroji Shinkawa:** data curation. **Yusuke Yamamoto:** data curation. **Masahiko Kinoshita:** data curation. **Koji Komeda:** data curation. **Tsukasa Aihara:** data curation. **Satoshi Yasuda:** data curation. **Haruki Mori:** data curation. **Masaki Kaibori:** supervision.

## Ethics Statement

This study was approved by the institutional review board of Kansai Medical University (Approval number: 2023320). It was performed in accordance with the Declaration of Helsinki.

## Conflicts of Interest

The authors declare no conflicts of interest.

## Supporting information


**Figure S1:** Effect of adjuvant chemotherapy on survival by lymph node status in stage IV intrahepatic cholangiocarcinoma. Overall survival is presented using Kaplan–Meier curves for stage IV intrahepatic cholangiocarcinoma, stratified by lymph node status (A: N0, B: N1). The green line represents patients who received adjuvant chemotherapy, and the blue line represents those who did not. Median overall survival is shown in months. AC, adjuvant chemotherapy; CI, confidence interval; mOS, median overall survival; NE, not estimable.

**Figure S2:** Effect of adjuvant chemotherapy on survival by IVA/IVB subclassification in stage IV intrahepatic cholangiocarcinoma. Overall survival is presented using Kaplan–Meier curves for stage IV intrahepatic cholangiocarcinoma, stratified by IVA/IVB subclassification (A: stage IVA, B: stage IVB). The green line represents patients who received adjuvant chemotherapy, and the blue line represents those who did not. Median overall survival is shown in months. AC, adjuvant chemotherapy; CI, confidence interval; mOS, median overall survival.

**Table S1:** Multivariable Cox regression analysis for overall survival in the entire cohort.
**Table S2:** Clinicopathological features of stage IVA and IVB intrahepatic cholangiocarcinoma.
**Table S3:** Clinicopathological features of stage IVA and IVB intrahepatic cholangiocarcinoma stratified by adjuvant chemotherapy status.
