# Supplementary figures and images for: Potential Survival Benefit of Adjuvant Chemotherapy in Stage IV Intrahepatic Cholangiocarcinoma: A Multicenter, Stage‐Stratified Analysis
Source: Ann Gastroenterol Surg. 2025 Aug 31;10(1):241–50. doi: 10.1002/ags3.70087 (PMC12757147; doi:10.1002/ags3.70087)

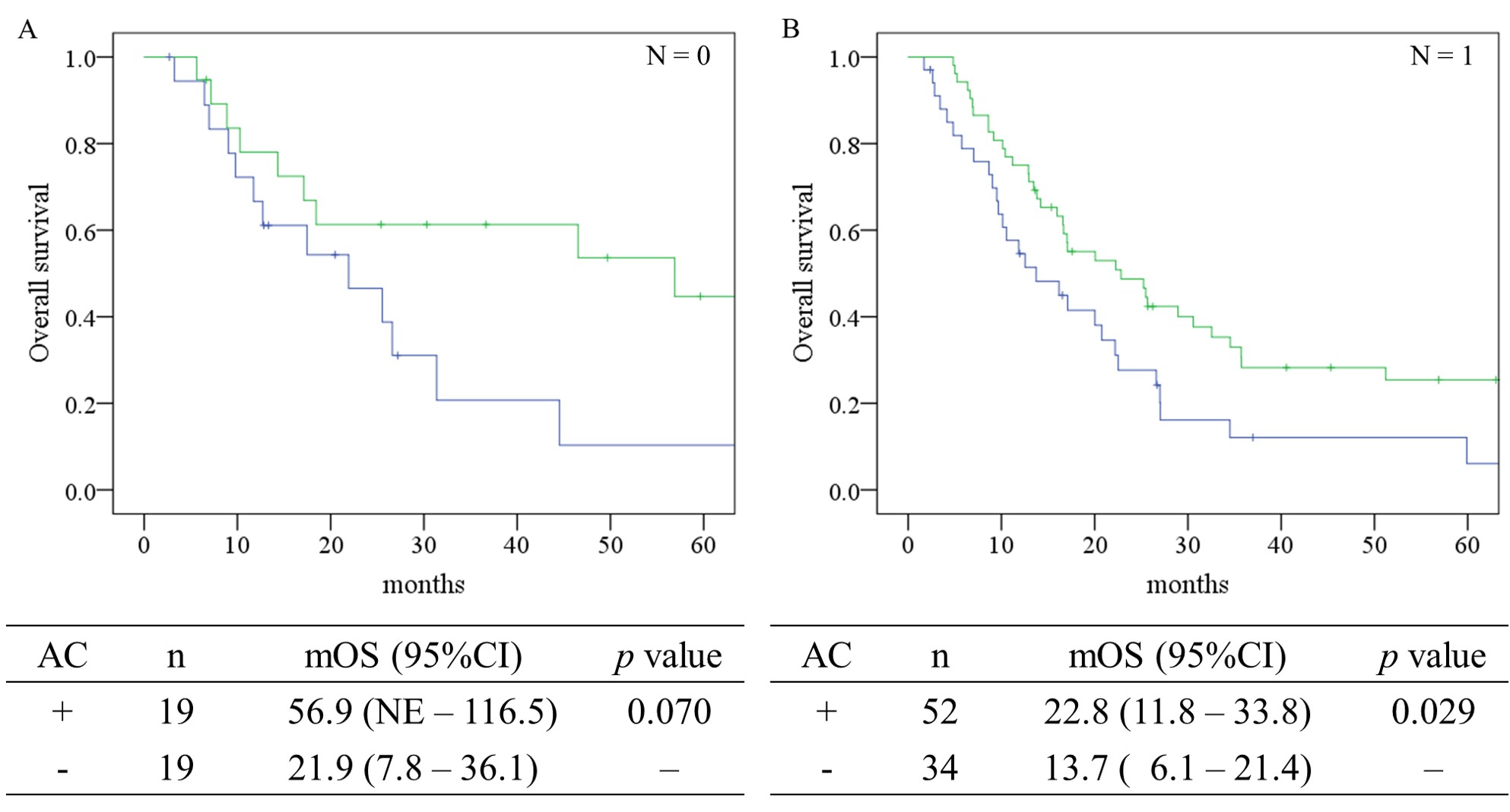

Supplement: Supplementary file 1 — Figure S1: Effect of adjuvant chemotherapy on survival by lymph node status in stage IV intrahepatic cholangiocarcinoma. Overall survival is presented using Kaplan–Meier curves for stage IV intrahepatic cholangiocarcinoma, stratified by lymph node status (A: N0, B: N1). The green line represents patients who received adjuvant chemotherapy, and the blue line represents those who did not. Median overall survival is shown in months. AC, adjuvant chemotherapy; CI, confidence interval; mOS, median overall survival; NE, not estimable. [file AGS3-10-241-s001.jpg]

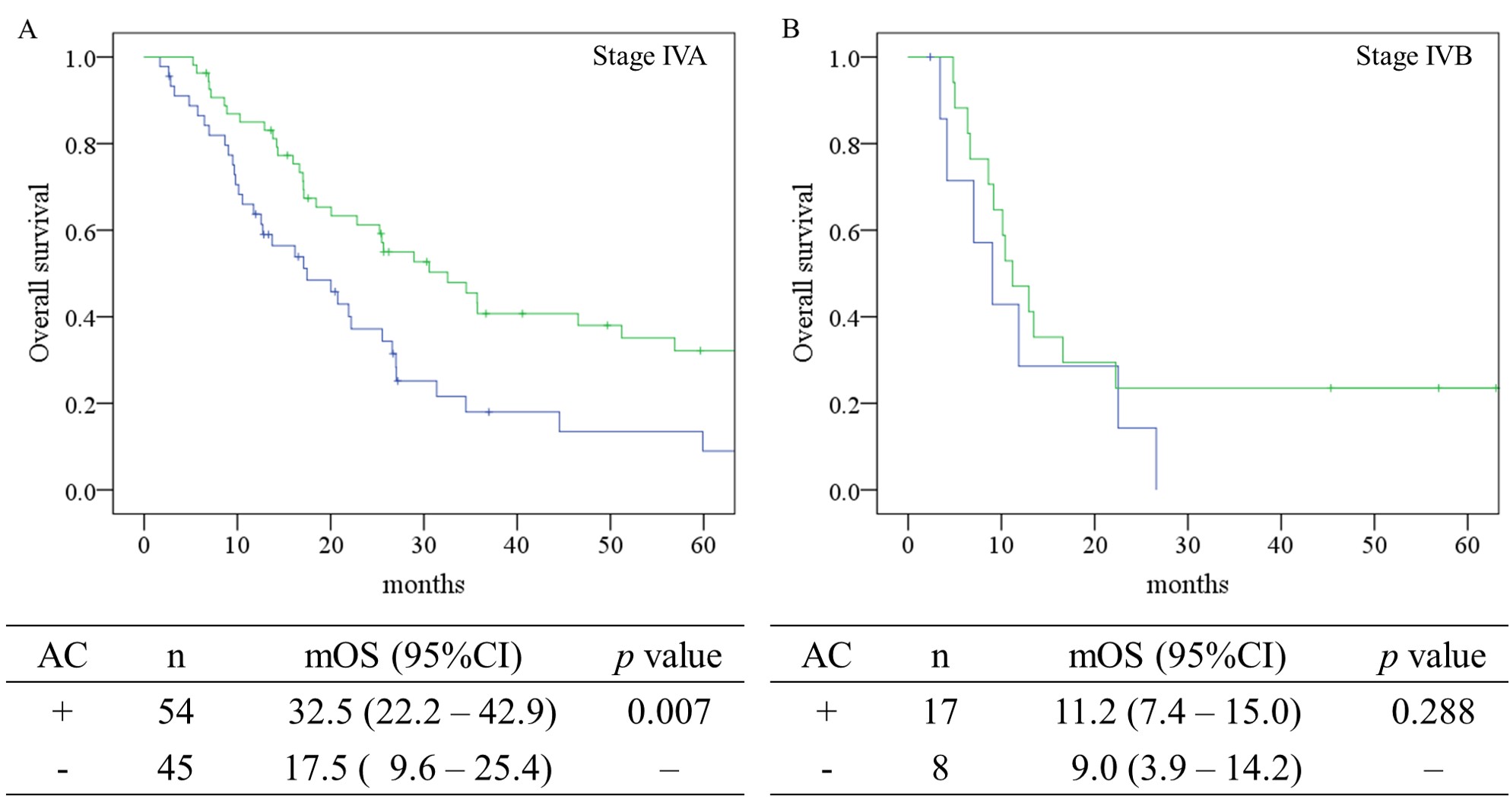

Supplement: Supplementary file 2 — Figure S2: Effect of adjuvant chemotherapy on survival by IVA/IVB subclassification in stage IV intrahepatic cholangiocarcinoma. Overall survival is presented using Kaplan–Meier curves for stage IV intrahepatic cholangiocarcinoma, stratified by IVA/IVB subclassification (A: stage IVA, B: stage IVB). The green line represents patients who received adjuvant chemotherapy, and the blue line represents those who did not. Median overall survival is shown in months. AC, adjuvant chemotherapy; CI, confidence interval; mOS, median overall survival. [file AGS3-10-241-s002.jpg]
