# Supplementary material for: Potential Survival Benefit of Adjuvant Chemotherapy in Stage IV Intrahepatic Cholangiocarcinoma: A Multicenter, Stage‐Stratified Analysis
Source: Ann Gastroenterol Surg. 2025 Aug 31;10(1):241–50. doi: 10.1002/ags3.70087 (PMC12757147; doi:10.1002/ags3.70087)
Supplement: Supplementary file 3 — Table S1: Multivariable Cox regression analysis for overall survival in the entire cohort. Table S2: Clinicopathological features of stage IVA and IVB intrahepatic cholangiocarcinoma. Table S3: Clinicopathological features of stage IVA and IVB intrahepatic cholangiocarcinoma stratified by adjuvant chemotherapy status. [file AGS3-10-241-s003.docx]

| **Supplementary Table 1. Multivariable Cox regression analysis for overall survival in the entire cohort** | | | | | | |
| --- | --- | --- | --- | --- | --- | --- |
| *Variable* | B | SE | Wald | *p* value | HR | 95% CI |
| *Age* | 0.001 | 0.008 | 0.021 | 0.885 | 1.001 | 0.985 – 1.017 |
| *Hepatitis C virus infection* | 0.015 | 0.225 | 0.004 | 0.948 | 1.015 | 0.652 – 1.579 |
| *ALBI score* | 0.502 | 0.199 | 6.387 | 0.011 | 1.652 | 1.119 – 2.438 |
| *FIB-4 index* | 0.047 | 0.039 | 1.441 | 0.230 | 1.048 | 0.971 – 1.132 |
| *CA19-9* | 0.298 | 0.061 | 24.200 | <0.001 | 1.347 | 1.196 – 1.516 |
| *Extent of hepatectomy* | -0.184 | 0.090 | 4.141 | 0.042 | 0.832 | 0.697 – 0.993 |
| *Clavien–Dindo ≥ IIIa* | 0.573 | 0.157 | 13.368 | 0.000 | 1.774 | 1.305 – 2.411 |
| *Resection margin status* | 0.198 | 0.185 | 1.136 | 0.286 | 1.218 | 0.847 – 1.752 |
| *Tumor diameter* | 0.010 | 0.003 | 14.124 | <0.001 | 1.010 | 1.005 – 1.016 |
| *Lymph node metastasis* | 0.939 | 0.165 | 32.217 | <0.001 | 2.558 | 1.849 – 3.537 |
| *Adjuvant chemotherapy* | -0.255 | 0.151 | 2.835 | 0.092 | 0.775 | 0.577 – 1.043 |
| Stage-specific analysis for patients with stage IV is presented in Table 3. B, regression coefficient; SE, standard error; Wald, Wald chi-square statistic; HR, hazard ratio; CI, confidence interval; ALBI, albumin–bilirubin score; FIB-4, fibrosis-4; CA19-9, carbohydrate antigen 19-9; | | | | | | |

| **Supplemental Table 2. Clinicopathological features of stage IVA and IVB intrahepatic cholangiocarcinoma** | | | |
| --- | --- | --- | --- |
| *Parameters* | n (%) or median (IQR) | | *p* value |
|  | *LCSGJ stage, IVA*  n = 99 | *LCSGJ stage, IVB*  n = 25 |  |
| *T stage*  *T1*  *T2*  *T3*  *T4* | 0 (0.0)  8 (8.1)  53 (53.5)  38 (38.4) | 0 (0.0)  0 (0.0)  5 (20.0)  20 (80.0) | 0.001 |
| *N stage*  *N0*  *N1* | 38 (38.4)  61 (61.6) | 0 (0.0)  25 (100.0) | <0.001 |
| *M stage*  *M0*  *M1* | 99 (100.0)  0 (0.0) | 24 (96.0)  1 (4.0) | 0.202 |
| *Resection margin*  *R0*  *R1* | 84 (84.8)  15 (15.2) | 16 (64.0)  9 (36.0) | 0.018 |
| IQR, interquartile range; LCSGJ, Liver Cancer Study Group of Japan; NE: not evaluable. | | | |

| **Supplemental Table 3. Clinicopathological features of stage IVA and IVB intrahepatic cholangiocarcinoma stratified by adjuvant chemotherapy status** | | | |
| --- | --- | --- | --- |
| *Parameters* | n (%) or median (IQR) | | *p* value |
|  | Adjuvant chemotherapy | No adjuvant chemotherapy |  |
| *LCSGJ stage, IVA* | n = 54 | n = 45 |  |
| *T stage*  *T1*  *T2*  *T3*  *T4* | 0 (0.0)  5 (9.3)  30 (55.6)  19 (35.2) | 0 (0.0)  3 (6.7)  23 (51.1)  19 (42.2) | 0.782 |
| *N stage*  *N0*  *N1* | 19 (35.2)  35 (64.8) | 18 (40.0)  27 (60.0) | 0.679 |
| *M stage*  *M0*  *M1* | 54 (100.0)  0 (0.0) | 45 (100.0)  0 (0.0) | NE |
| *Resection margin*  *R0*  *R1* | 46 (85.2)  8 (14.8) | 38 (84.4)  7 (15.6) | 1.000 |
| *LCSGJ stage, IVB* | n = 17 | n = 8 |  |
| *T stage*  *T1*  *T2*  *T3*  *T4* | 0 (0.0)  0 (0.0)  4 (23.5)  13 (76.5) | 0 (0.0)  0 (0.0)  1 (12.5)  7 (87.5) | 0.782 |
| *N stage*  *N0*  *N1* | 0 (0.0)  17 (100.0) | 0 (0.0)  8 (100.0) | NE |
| *M stage*  *M0*  *M1* | 16 (94.1)  1 (5.9) | 8 (100.0)  0 (0.0) | 0.680 |
| *Resection margin*  *R0*  *R1* | 11 (64.7)  6 (35.3) | 5 (62.5)  3 (37.5) | 1.000 |
| IQR, interquartile range; LCSGJ, Liver Cancer Study Group of Japan; NE: not evaluable. | | | |
